# Endocrine-disrupting pesticides and breast cancer incidence in the United States: an ecological analysis

**DOI:** 10.1007/s10552-026-02188-3

**Published:** 2026-06-01

**Authors:** Harish Neelam, Furqan Irfan, Robert Wahl, Kelly A. Hirko

**Affiliations:** 1 Department of Epidemiology and Biostatistics, College of Human Medicine, Michigan State University, East Lansing, MI USA; 2 Department of Neurology and Ophthalmology, College of Osteopathic Medicine, Michigan State University, East Lansing, MI USA; 3 Charles Stewart Mott Department of Public Health, College of Human Medicine, Michigan State University, East Lansing, MI USA

**Keywords:** Pesticides, Breast cancer, Environmental health, Ecologic, Endocrine

## Abstract

**Purpose:**

We examined the associations between county-level endocrine-disrupting pesticide usage and breast cancer incidence, overall and by metropolitan status.

**Methods:**

This ecological study used publicly available data from the United States Geological Survey on county-level agricultural pesticide usage (in kilograms). We focused on 38 pesticides with endocrine-disrupting properties relevant in breast cancer etiology, which were categorized by chemical class as follows: carbamates, neonicotinoids, organochlorines, organophosphates, phosphonates, pyrethroids, triazines, and other miscellaneous compounds. The analysis included 2,457 U.S. counties with data on pesticide usage (2001–2015) and age-adjusted breast cancer incidence rates (2016–2020), accounting for a latency period. Metropolitan status was assessed using the 2013 Rural–Urban Continuum Codes. Gamma regression models with a log-link function were used to estimate rate ratios (RR) and 95% confidence intervals (CI) between pesticides (per interquartile range increase) and age-adjusted breast cancer incidence rates, adjusting for county-level covariates including % smoking, % poverty, % unemployed, % no high school diploma, % uninsured, and residential mobility.

**Results:**

Total median pesticide use was higher in rural vs. urban counties (17,523 kg vs. 14,743 kg; *p* < 0.05), with some variation by pesticide class. Pesticide use was associated with slightly higher breast cancer incidence in rural counties (RR = 1.02 (95% CI 1.01, 1.03)), but not in urban counties (RR = 1.00 (95% CI 1.00, 1.00)) (*p*_int_ < 0.001). Associations differed by pesticide type.

**Conclusion:**

Results suggest modest positive associations between county-level endocrine-disrupting pesticide use and breast cancer incidence in rural U.S. counties, with variation by pesticide type. These findings highlight the need for further research to inform targeted prevention efforts.

**Supplementary Information:**

The online version contains supplementary material available at 10.1007/s10552-026-02188-3.

## Introduction

Breast cancer remains the most common cancer among women in the United States (U.S.) [1], with an estimated 316,950 new cases expected in 2025 [2]. Over the past decade, U.S. breast cancer incidence rates have risen by 1% annually, with a slightly steeper increase among women diagnosed before age 50 years (1.4% per year) [2]. Breast cancer incidence rates are higher in urban than in rural U.S. counties [3], likely due in part to lower screening rates in rural areas [4] and have risen over time in both settings [3]. Trends in breast cancer incidence differ by estrogen receptor (ER) status, with rising rates of ER-positive and slightly declining rates of ER-negative breast cancers over time [5].

Changes in the prevalence of breast cancer risk factors, including excess body weight and reproductive factors, may contribute to rising breast cancer incidence rates [6]. However, the role of environmental factors, particularly exposures to endocrine-disrupting chemicals, warrants further investigation. Indeed, many commonly used agricultural pesticides [7] have demonstrated carcinogenic potential through endocrine disruption pathways relevant to breast cancer etiology [8–11]. Given the biological plausibility of a link between pesticide exposure and breast cancer risk, and the increasing use of pesticides over time [12], endocrine-disrupting pesticides may contribute to rising breast cancer rates.

Epidemiologic research examining the link between pesticide exposure and breast cancer risk has shown some positive associations, though findings remain inconsistent across studies [13]. A systematic review of 131 observational studies concluded that exposure to endocrine-disrupting chemicals, including pesticides, may increase breast cancer risk, but evidence was insufficient to establish causality [14]. Previous studies have been limited by methodological challenges, such as inadequate consideration of the latency period between exposure and diagnosis [10], and limited assessment of variability across pesticide types and geographic regions [14]. Further prospective research is needed to clarify the role of endocrine-disrupting pesticides in breast cancer risk.

The aim of this study was to examine the associations between county-level use of pesticides classified as endocrine-disrupting chemicals and breast cancer incidence rates, overall and based on county metropolitan status, while accounting for potential latency between exposure and breast cancer diagnosis.

## Methods

### Study design and population

We conducted an ecological study examining associations between county-level endocrine-disrupting pesticide use and age-adjusted breast cancer incidence rates. To account for the average latency period of 5–15 years between pesticide exposure and breast cancer diagnosis [15], we used pesticide data from 2001 to 2015 and the 5-year breast cancer incidence rates from 2016 to 2020. Of the total 3,143 U.S. counties, we excluded from analysis 77 counties with missing information on pesticides and 609 counties with missing information on breast cancer incidence, resulting in a final analytic sample of 2,457 counties (Supplementary Fig. 1).

### Primary exposure

Data on county-level pesticide use were obtained from the U.S. Geological Survey Pesticide National Synthesis Project [16] and included information on the type of pesticide used, the county and state of usage, the year of use, and the amount used in kilograms. The database included information on approximately 500 pesticides from years 1992 to 2017. County-level pesticide use was estimated using the “EPest-high” measures. These estimates reflect agricultural applications and do not capture residential or other non-agricultural uses. Briefly, harvested-crop acreage data by county from the U.S. Department of Agriculture Census of Agriculture are used to calculate the median pesticide-by-crop use rates for each crop in each Crop Reporting District (CRD). Rates from neighboring or regional CRDs are used to impute pesticide-by-crop rates for CRDs with missing data. These rates are applied to the harvested acreage of each crop in a county to obtain pesticide use estimates at the county-level [17, 18]. The USGS Pesticide National Synthesis Project does not classify pesticides according to endocrine-disrupting properties. Therefore, we identified pesticides with endocrine-disrupting activity relevant in breast cancer etiology based on prior toxicologic and epidemiologic literature [19–21]. We calculated county-level cumulative average agricultural pesticide usage from 2001 to 2015 for 38 chemical pesticides with endocrine disruption properties. We grouped these 38 pesticides into the following 8 classes based on their chemical structure [22], shown in Table 1: carbamates, neonicotinoids, organochlorines, organophosphates, phosphonates, pyrethroids, triazines, and other miscellaneous. Organochlorines, triazines, and organophosphates have the strongest evidence for endocrine activity, whereas carbamates, pyrethroids, neonicotinoids, and phosphonates have more limited or emerging evidence of endocrine-disrupting effects [19–21]. We used continuous measures of endocrine-disrupting pesticides in kilograms and categorized overall use in tertiles and per interquartile range increase for analysis.

**Table 1:** Average annual total of pesticides with endocrine disruption properties used in kilograms over 15 years (2001 – 2015) in the U.S. counties, median (interquartile range; IQR), and range

Pesticide class	Compounds	Overall U.S. *N* = 2,457	Rural counties *N* = 1,472	Urban counties *N* = 985	*p*-value^a^
		Median (IQR) range	Median (IQR) range	Median (IQR) range	
Carbamates	Aldicarb Carbaryl Lindane Propoxur	234 (547) 0.1 – 47,992	242 (565) 0.1 – 47,992	229 (524) 0.2 – 30,663	0.60
Neonicotinoids	Acetamiprid Clothianidin Dinotefuran Imidacloprid Thiacloprid Thiamethoxam	152 (573) 0 – 26,409	166 (631) 0 – 5,258	138 (455) 0 – 26,409	0.19
Organochlorines	Dicofol Endosulfan Methoxychlor	27 (95) 0.1 – 26,652	21 (78) 0.1 – 6,897	37 (125) 0.1 – 26,652	< 0.001
Organophosphates	Azinphos-Methyl Bensulide Chlorpyrifos Diazinon Dicrotophos Dimethoate Disulfoton Ethoprophos Fenamiphos Malathion Naled Parathion Phorate	1,268 (2,887) 0.1 – 200,913	1,395 (3,067) 0.2 – 84,365	1,181 (2,713) 0.1 – 200,913	0.37
Phosphonates	Glufosinate Glyphosate	8,483 (39,426) 0.1 – 466,037	9,638 (49,193) 0.6 – 315,970)	7,503 (28,805) 0.1 – 466,037	0.01
Pyrethroids	Cypermethrin Fenpropathrin Permethrin	56 (161) 0 – 17,556	54 (164) 0 – 4,168	57 (156) 0 – 17,556	0.40
Triazines	Atrazine Propazine Simazine	2,625 (12,491) 0.1 – 568,260	2,606 (14,579) 0.1 – 148,710	2,659 (10,275) 1.4 – 568,260	0.53
Miscellaneous	Fosetyl Terbufos Tribufos Triclopyr	492 (984) 0 – 88,464	525 (1,031) 0.2 – 47,880	418 (903) 0 – 88,464	0.01
Total Pesticides^b^		15,947 (64,379) 0.1 – 865,154	17,523 (77,162) 1.0 – 521,102)	14,743 (48,816) 0.1 – 865,154	0.02

*IQR* interquartile range

^a^Wilcoxon rank-sum test was used to compare non-normal continuous pesticide usage between rural and urban counties

^b^Total pesticides represents 38 endocrine-disrupting pesticides categorized into 8 classes

### Primary outcome

The most recent 5-year average age-adjusted breast cancer incidence rates among female residents for U.S. counties at the time of analysis were ascertained from the National Cancer Institute's State Cancer Profiles (2016–2020) [23]. Breast cancer incidence rates (cases per 100,000 women per year) were age-adjusted to the 2000 U.S. standard population. Breast cancer incidence rates were calculated for 2,479 counties and county equivalents in the U.S.; data were not reported for counties in Minnesota, Virginia, Kansas, and Nevada and for 212 counties with suppressed data due to insufficient case counts. We assumed a 5–15 latency between cumulative pesticide exposure (2001–2015) and breast cancer rates (2016–2020), consistent with prior epidemiologic evidence that breast cancer development following environmental and endocrine-disrupting exposures occurs over multiple years [10, 24]. This window captures a biologically plausible etiologic period, while minimizing potential exposure misclassification due to residential mobility and changes in county-level pesticide use over time.

#### Covariates and stratifying variables

We obtained county-level data on the following potential covariates based on prior knowledge of breast cancer risk factors: smoking prevalence, poverty, unemployment, educational attainment, and adult health insurance coverage. We also assessed the proportion of residents living in the same house one year prior as a measure of residential mobility, obtained from the American Community Survey, in the multivariable model. Data for smoking prevalence were obtained from county health rankings and roadmaps, a program managed by the University of Wisconsin population health institute [25]. Other covariate data were obtained from the U.S. Centers for Disease Control and Prevention, Agency for Toxic Substances and Disease Registry, & Geospatial Research, Analysis & Services Program [26]. All covariate data reflected 5-year average estimates from 2016 to 2020, aligned with the outcome data. In stratified analysis, counties were classified into rural or urban based on the county-level 2013 rural–urban continuum codes (RUCC) obtained from the USDA website [27]. Counties with a RUCC code of 1–3 were considered urban, while counties with a RUCC code of 4–9 were considered rural.

### Statistical analysis

Descriptive statistics were used to summarize pesticide use by chemical class, overall and by county metropolitan status. Differences in pesticide usage by metropolitan status were assessed using Wilcoxon–Mann–Whitney tests. We used simple linear regression and the Mann–Kendall test to evaluate trends in usage of pesticides with endocrine disruption properties over time from 2001 to 2015, overall and by county metropolitan status. We examined county characteristics across tertiles of total endocrine-disrupting pesticide use and used linear regression to calculate a p-value for linear trend. We created county-level choropleth maps to visualize the spatial distribution of the 38 pesticides’ usage and age-adjusted breast cancer incidence rates across the United States.

To examine the associations between endocrine-disrupting pesticide use and age-adjusted breast cancer incidence rates, we employed gamma regression with a log-link function to estimate the rate ratio (RR; exp(β)) and its 95% confidence interval (CI). County-level age-adjusted breast cancer incidence rates, which were positive and right-skewed, were modeled using gamma regression with a log link to account for non-normality and heteroscedasticity while providing interpretative multiplicative effects. We assessed residual spatial autocorrelation using Moran’s I based on county adjacency and found it to be negligible; therefore, a non-spatial regression framework was used. Given the potential for localized spatial heterogeneity, we further explored spatial patterns using bivariate Local Indicators of Spatial Association (LISA) to examine county-level relationships between pesticide use and breast cancer incidence. Local spatial clusters (high–high, low–low, high–low, and low–high) were identified based on spatially weighted relationships between the pesticide classes and age-adjusted breast cancer incidence rates. Statistical significance was assessed using Monte Carlo permutation testing.

The multivariable model was adjusted for average % smoking, % below 150% of the poverty level, % unemployed, % no high school diploma, % uninsured, and the % of residents living in the same house one year prior as a measure of residential mobility. We assessed multicollinearity using variance inflation factors to ensure stable coefficient estimates in the multivariable gamma regression models. We further evaluated models stratified by county metropolitan status and assessed differences by rurality using the Wald test for the interaction term. Given the differing distributions of pesticides, we report the estimated associations per interquartile range (IQR) increase to facilitate interpretation and comparison across pesticide groups.

To assess potential selection bias, we compared county-level characteristics between counties included in the analytic sample and those excluded due to missing data, reporting medians and inter quartile ranges, Cohen’s d effect sizes, and corresponding p-values. In secondary analyses, we assessed associations based on tertiles of pesticide use, for each individual pesticide type, and using the more conservative EPA estimate of county-level pesticides using lower-bound assumptions (EPest low). Given the rising rates of young-onset breast cancer in the U.S., we also explored associations between pesticides and breast cancer rates among women < 50 years of age in secondary analysis. Two-sided *p*-values < 0.05 were considered statistically significant. Data cleaning, processing, and statistical analyses were conducted using R software (version 4.5.1).

## Results

As shown in Table 1, median annual endocrine-disrupting pesticide use was higher in rural vs. urban counties (17,523 kg vs. 14,743 kg; p = 0.02), with some variation by pesticide class. The use of phosphonates (9,638 kg vs. 7,503 kg; *p* = 0.01) and other miscellaneous pesticides (525 kg vs. 418 kg; *p* = 0.01) was higher in rural vs. urban U.S. counties. In contrast, the use of organochlorines (21 kg vs. 37 kg; *p* < 0.001) was lower in rural compared to urban counties. Use of carbamates, neonicotinoids, organophosphates, pyrethroids, and triazines did not differ significantly across rural and urban U.S. counties. Phosphonates (glufosinate and glyphosate marketed as Roundup®) were the most heavily used endocrine-disrupting pesticides with a median 8,483 kg applied per year, followed by triazines (atrazine, propazine and simazine) at a median of 2,625 kg annually.

**Fig. 1:**
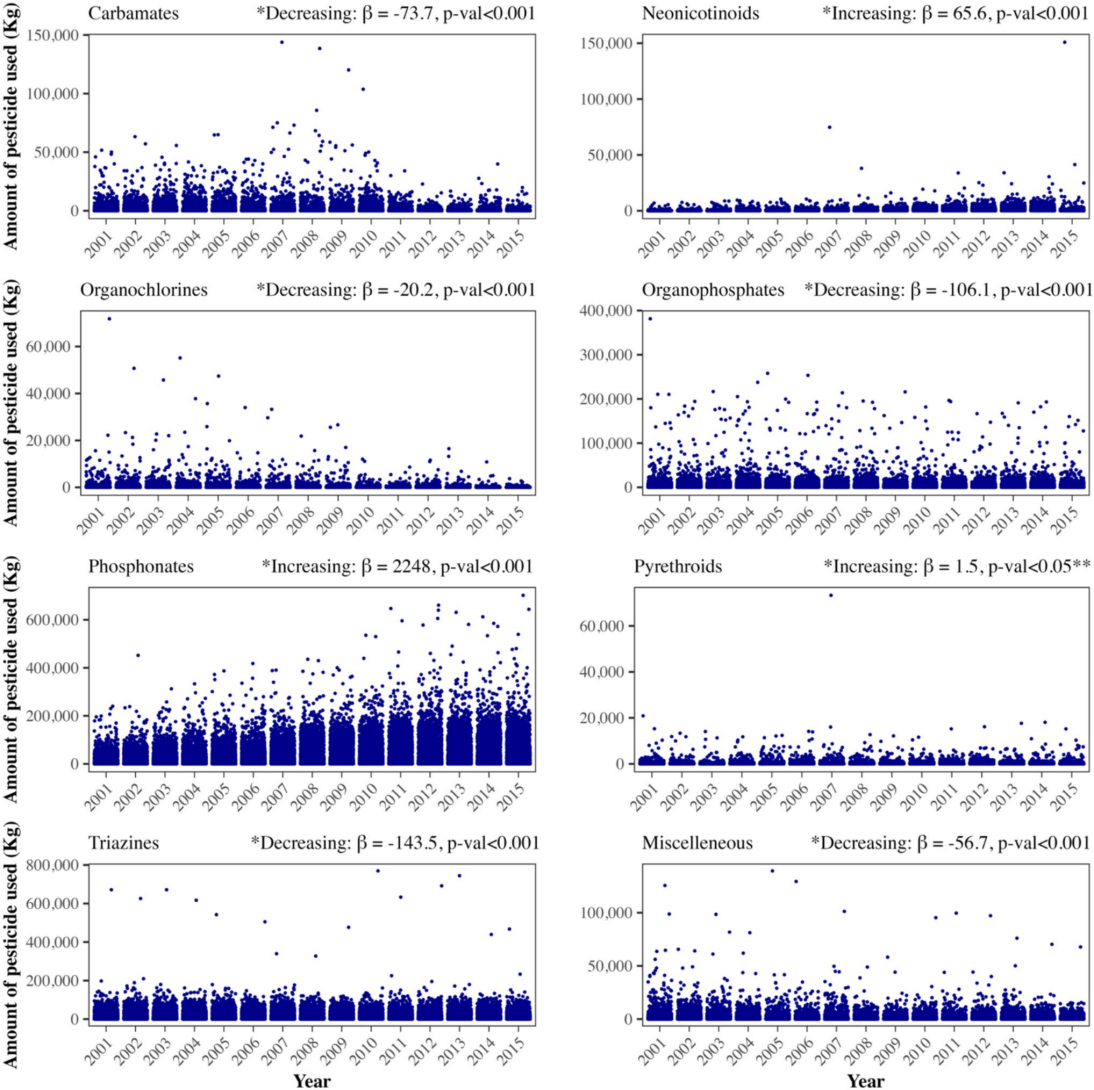
Trends in the amount of pesticides with endocrine-disrupting properties used (Kg) from 2001 to 2015 across U.S. Counties by pesticide class. *Beta estimates and *p*-values obtained from the linear regression model assessing average annual pesticide use over time; **Significant non-monotonic trend; the Mann–Kendall test was not significant

Trends in endocrine-disrupting pesticides over time are shown in Fig. 1. The use of carbamates (β = − 73.7,), organochlorines (β = − 20.2), organophosphates (β = − 106.1), triazines (β = − 143.5), and other miscellaneous pesticides (β = − 56.7) decreased significantly over time from 2001 to 2015 (all *p*_trend_ < 0.001). However, increasing trends were observed for neonicotinoids (β = 65.6, *p*_trend_ < 0.001), phosphonates (β = 2248, *p*_trend_ < 0.001), and pyrethroids (β = 1.5, *p*_trend_ < 0.05) over time. Similar trends were observed in analyses stratified by rurality (Supplementary Fig. 2).

The geographic distribution of endocrine-disrupting pesticide usage (2001–2015) and age-adjusted breast cancer incidence rates from 2016 to 2020 in the U.S. are shown in Supplementary Fig. 3. Endocrine-disrupting pesticide use was higher in the central northern region, with smaller areas of higher use in California, Washington, Montana, and Florida. The geographic distribution of age-adjusted breast cancer incidence rates from 2016 to 2020 was more dispersed, with some areas of higher rates in the central northern region and lower incidence rates in the southwestern U.S. counties.

County-level characteristics varied across tertiles of endocrine-disrupting pesticide use (Table 2). The average percentages of smoking, below 150% poverty, unemployment, and uninsurance all decreased with increasing pesticide tertile (all *p*_trend_ < 0.05). Age-adjusted breast cancer incidence rates were higher in counties with higher pesticide use (123.7 vs. 119.4 per 100,000 in Tertile 3 vs. Tertile 1; *p*_trend_ < 0.05).

**Table 2:** Characteristics of 2,457 U.S. counties (2016 – 2020) by total pesticide use (kg) of 38 pesticides with endocrine disruption properties applied from 2001 to 2015

	Total pesticides (Kg)*			*p*-value_trend_
Measure	Tertile 1 (Low) < 5,600 *N* = 816	Tertile 2 (Medium) ≥5,600 & < 42,000 *N* = 832	Tertile 3 (High) ≥42,000 *N* = 809	
	Mean (SD)	Mean (SD)	Mean (SD)	
Age-adjusted breast cancer incidence rate per 100,000	119.4 (19.9)	120.9 (19.8)	123.7 (20.7)	0.01
% Smoking^a^	18.4 (3.9)	18.1 (3.3)	17.8 (3.3)	< 0.001
% Poverty^b^	20.2 (6.9)	19.6 (6.6)	18.6 (7.1)	0.01
% Unemployed^c^	6.8 (2.2)	6.5 (2.3)	5.7 (2.7)	< 0.001
% No high school diploma^d^	13.9 (6.0)	14.0 (5.7)	13.1 (6.3)	0.20
% Uninsured^e^	10.9 (4.4)	11.2 (4.7)	9.7 (5.0)	< 0.001
% Residential mobility^f^	12.5 (4.1)	12.7 (3.9)	12.7 (4.0)	0.20

*SD* standard deviation

*p*-value_trend_, linear trend *p*-value for measure with increasing pesticide use

^*^Total pesticide usage represents the county-level average of annual total use of 38 pesticides over the period of 2001–2015 and is referred to consistently throughout the manuscript

^a^Proportion of adults in the county that report current smoking

^b^Percentage of the county population living below 150% of the federal poverty line

^c^Percentage of the county’s labor force (age 16 + civilians) that is actively looking for work but is currently unemployed

^d^Proportion of adults (age 25 +) in the county with no high school diploma

^e^Percentage of adults uninsured in the total noninstitutionalized population

^f^Percentage of residents living in a different residence one year prior

Age-adjusted and multivariable-adjusted associations of breast cancer incidence per IQR increase in county-level pesticide use in the overall U.S. and by rurality are shown in Table 3. Endocrine-disrupting pesticide use was positively associated with breast cancer incidence rates in rural counties. Results were similar, though slightly attenuated in multivariable models. After adjusting for covariates, endocrine-disrupting pesticide use was positively associated with breast cancer incidence in rural (aRR = 1.02,95% CI 1.01–1.03), but not in urban (aRR = 1.00, 95% CI 1.00–1.00) counties (*p*_int_ < 0.001). Associations differed by pesticide type and by metropolitan status. In multivariable models, neonicotinoids (aRR = 1.01, 95% CI 1.01–1.02) and phosphonates (aRR = 1.02, 95% CI 1.01–1.03) were associated with higher breast cancer rates in rural counties but associations were not significant in urban regions (both *p*_int_ < 0.05). Carbamates, organochlorines, organophosphates, pyrethroids, triazines, and miscellaneous endocrine-disrupting pesticides were not significantly associated with breast cancer rates in multivariable models.

**Table 3:** Age-adjusted and multivariable-adjusted associations between breast cancer incidence and interquartile range (IQR) increase in county-level pesticide use in the U.S., overall and by rurality

Pesticide use (Kg) (2001 – 2015)	Age-adjusted breast cancer rates (2016 – 2020)						
	Overall U.S *N* = 2,457		Rural counties *N* = 1,472		Urban counties *N* = 985		*p*-value_int_
	Age-adjusted $RR$* (95% CI)	Multivariable $aRR$** (95% CI)	Age-adjusted $RR$* (95% CI)	Multivariable $aRR$** (95% CI)	Age-adjusted $RR$* (95% CI)	Multivariable $aRR$** (95% CI)	
Total pesticide	1.01 [1.00, 1.02]	1.01 [1.00, 1.01]	1.03 [1.02, 1.04]	1.02 [1.01, 1.03]	1.00 [1.00, 1.00]	1.00 [1.00, 1.00]	< 0.001
Carbamates	1.00 [1.00, 1.00]	1.00 [1.00, 1.00]	1.00 [1.00, 1.00]	1.00 [1.00, 1.01]	1.00 [1.00, 1.00]	1.00 [1.00, 1.00]	0.001
Neonicotinoids	1.00 [1.00, 1.01]	1.00 [1.00, 1.01]	**1.02** **[1.01, 1.03]**	**1.01** **[1.01, 1.02]**	1.00 [1.00, 1.00]	1.00 [1.00, 1.00]	< 0.001
Organochlorines	1.00 [1.00, 1.00]	1.00 [1.00, 1.00]	1.00 1.00, 1.00]	1.00 [1.00, 1.01]	**0.96** **[0.95, 0.98]**	0.98 [0.97, 1.00]	< 0.001
Organophosphates	1.00 1.00, 1.00]	1.00 [1.00, 1.00]	1.00 [1.00, 1.01]	1.01 [1.00, 1.02**]**	**0.97** **[0.95, 0.98]**	0.98 [0.97, 1.00]	0.002
Phosphonates	1.01 [1.00, 1.02]	1.01 [1.00, 1.01]	**1.03** **[1.02, 1.04]**	**1.02** **[1.01, 1.03]**	1.00 [1.00, 1.00]	1.00 [1.00, 1.00]	0.001
Pyrethroids	1.00 [1.00, 1.00]	1.00 [1.00, 1.00]	1.00 [1.00, 1.01]	1.01 [1.00, 1.02]	**0.84** **[0.77, 0.92]**	0.92 [0.84, 1.02]	0.009
Triazines	**1.01** **[1.01, 1.01]**	1.00 [1.00, 1.01]	**1.02** **[1.01, 1.03]**	1.01 [1.00, 1.02]	1.00 [1.00, 1.00]	1.00 [1.00, 1.00]	0.094
Miscellaneous	1.00 [1.00, 1.00]	1.00 [1.00, 1.01]	1.00 [1.00, 1.00]	1.01 [1.00, 1.01]	1.00 [1.00, 1.00]	1.00 [1.00, 1.00]	0.002

*CI* confidence interval

*p*-value_int_, rural–urban interaction p-value after adjusting for covariates;

^*^$RR$ = Rate ratio per IQR increase in pesticide use

^**^$aRR$ = Adjusted rate ratio per IQR increase in pesticide use; multivariable model adjusted for % smoking, % poverty, % unemployed, % no high school diploma, % uninsured, and % residential mobility

Bolded numbers represent statistically significant associations (P<0.05)

Moran’s I values ranged from − 0.008 to 0.025, indicating minimal spatial autocorrelation in the models and suggesting no meaningful residual spatial dependence. Local Indicators of Spatial Association (LISA) analyses (Supplementary Fig. 4) were used to explore local spatial associations between breast cancer incidence and endocrine-disrupting pesticide use. Statistically significant clusters were limited and did not form consistent or contiguous spatial patterns. A small number of high–-high clusters (counties with elevated age-adjusted breast cancer rates surrounded by high pesticide use) and low–high clusters were identified, but these were geographically dispersed. Overall, the observed spatial patterns were weak and heterogeneous, consistent with minimal global spatial autocorrelation.

Most county-level characteristics and pesticide usage for counties included in the analysis (*n* = 2,457) differed significantly from those excluded due to missing data (*n* = 686), though differences were small in magnitude (Supplementary Table 1). In a supplemental analysis assessing tertiles of total endocrine-disrupting pesticide use in relation to breast cancer incidence (Supplementary Table 2), results were generally consistent with the main findings. Higher pesticide use was associated with modestly increased age-adjusted breast cancer rates overall, particularly in the highest tertile of exposure (aRR_T3 vs. T1_ = 1.03, 95% CI 1.01–1.04). Stratified analysis indicated that this association was driven by rural counties, where high pesticide use was associated with higher breast cancer incidence rates (aRR_T3 vs. T1_ = 1.06, 95% CI 1.03–1.08), while no association was observed in urban counties (aRR_T3 vs. T1_ = 0.99, 95% CI 0.97–1.01). In supplemental analyses of individual pesticide types (Supplementary Table 3), most associations were null, though interpretation was limited by small sample sizes for several pesticides. In rural counties, thiamethoxam (aRR = 1.02, 95% CI 1.01–1.02), chlorpyrifos (aRR = 1.01, 95% CI 1.01–1.02), glufosinate (aRR = 1.01, 95% CI 1.01–1.02), and glyphosate (aRR = 1.02, 95% CI 1.01–1.03) were modestly associated with higher breast cancer rates.

In supplemental analyses using lower-bound EPA pesticide estimates (Supplementary Table 4), associations were generally consistent with the main findings. Total pesticide use was associated with higher breast cancer rates in rural counties (aRR = 1.02, 95% CI 1.01–1.03). By pesticide class, most associations were null or very small; however, phosphonates were associated with higher breast cancer rates in rural counties (aRR = 1.02, 95% CI 1.01–1.03). While secondary analysis of young-onset breast cancer was limited by smaller sample size (*n* = 1,405 U.S. counties with estimated young-onset breast cancer rates), we did not observe any significant associations between endocrine-disrupting pesticides and young-onset breast cancer incidence rates (Supplementary Table 5).

## Discussion

Findings from this ecological study suggest modest positive associations between county-level endocrine-disrupting pesticide use and breast cancer incidence rates in rural U.S. counties, with variation by pesticide type. This pattern was consistent across multiple sensitivity analysis, including alternative exposure metrics and categorical exposure models. Among pesticide classes, neonicotinoids and phosphonates were most consistently associated with higher breast cancer rates in rural settings, though the magnitude of associations was small. Overall, pesticide use was higher in rural counties than in urban counties, and the use of several pesticides linked to increased breast cancer rates in rural areas has been steadily increasing over time (i.e., neonicotinoids and phosphonates). The rising use of these pesticides suggests a growing burden of cumulative exposure to endocrine-disrupting chemicals, underscoring the importance of enhanced monitoring and surveillance.

Consistent with a prior meta-analysis [28] and systematic review [13], our findings suggest heterogeneity in associations between endocrine-disrupting pesticides and breast cancer by pesticide class. In this study, positive associations were observed primarily for neonicotinoids and phosphonates, while no significant associations were identified for organochlorines or organophosphates. These findings differ from a prior meta-analysis reporting positive associations between organochlorines and breast cancer rates [28]. However, the results across the literature remain inconsistent. For example, a study conducted in an agricultural region of California observed no association between organochlorine pesticide exposure and breast cancer; however, elevated risk was observed for specific organophosphates, including chlorpyrifos [29]. In contrast, organophosphates were not significantly associated with breast cancer in our study. However, in a secondary analysis of individual pesticide types, chlorpyrifos was associated with slightly higher breast cancer rates in rural counties. Our findings of positive associations between neonicotinoids [30], driven by thiamethoxam, and phosphonates, [31], including glufosinate and glyphosate, with breast cancer risk are biologically plausible given effects on pathways relevant in breast cancer etiology [30]. However, epidemiologic evidence for the role of these pesticides on breast cancer remains limited, inconsistent, and inconclusive [32, 33]. These inconsistencies likely reflect challenges in accurately measuring pesticide exposure, including limited information on timing, duration, and dose. Thus, while the evidence suggests possible links between exposures to specific pesticides and breast cancer risk, more rigorous research is needed.

Interestingly, some pesticide classes with well-established endocrine-disrupting activity, such as organophosphates, were not associated with breast cancer incidence in this study. This may reflect differences in patterns of agricultural use, exposure routes, or environmental persistence, which could result in lower cumulative population-level exposure despite strong mechanistic evidence. Conversely, observed associations for pesticides with more moderate or limited evidence of endocrine activity, such as neonicotinoids and phosphonates, [19, 20] suggest that additional biological pathways beyond endocrine disruption may be relevant. Organochlorines, triazines, and organophosphates have been shown to alter estrogen and androgen signaling, potentially influencing hormone-dependent breast tissue growth [19, 20]. Experimental and epidemiologic studies further suggest that these compounds can induce oxidative stress and DNA damage, disrupt key cell signaling pathways involved in proliferation and apoptosis, and promote epigenetic modifications such as altered DNA methylation in breast tissue [34, 35]. Future studies incorporating individual-level exposure assessment and biomarkers of estrogenic activity are needed to clarify the role of these biologic pathways in breast cancer etiology.

Our finding showed that the associations between endocrine-disrupting pesticides and breast cancer rates were significant only in rural counties, even when the use of certain pesticides was higher in urban areas, which suggests that aspects of the rural environment may heighten exposure or susceptibility. Although phosphonates were used in the greatest quantities across both rural and urban counties, they were associated with higher breast cancer rates only in rural areas. Likewise, neonicotinoids were used far less overall and at only slightly higher levels in rural counties, yet their associations with breast cancer were also evident only in rural counties. These patterns suggest that factors beyond total application volume, such as proximity to agricultural fields, occupational exposures, reliance on private wells, or other factors related to rural ecosystems, may increase population-level exposure in rural regions [36].

Findings from the exploratory analysis of spatial clustering using LISA indicated limited evidence of spatial structure in the relationship between endocrine-disrupting pesticide use and breast cancer incidence. Most counties did not exhibit statistically significant local spatial autocorrelation, and the significant clusters identified were sparse and geographically scattered. Although some isolated high–high and low–high clusters were significant, these were not consistent across pesticide class or region and did not form coherent spatial patterns. Overall, these findings suggest that any spatial association between pesticide use and breast cancer incidence is weak, inconsistent, and highly localized. As such, the results should be interpreted cautiously and primarily as exploratory, without strong evidence of widespread or stable geographic clustering.

Strengths of this study include the consideration of the latency period between exposure to pesticides and breast cancer diagnosis. Moreover, our focus on endocrine-disrupting pesticides that are biologically linked to breast cancer enhances the relevance of our findings. By using county-level data, the study provides a comprehensive overview of pesticide use patterns and their associations with breast cancer incidence across the United States. Importantly, our study was the first to our knowledge to assess variation in associations across rural and urban counties, offering valuable insights into how geographic context shapes expose risk and potential disparities in health outcomes. Despite these strengths, the study has important limitations. First, reliance on county-level data introduces the potential for ecological bias, and our findings cannot be directly inferred at the individual level. Our county-level measure of pesticide exposure is imprecise and does not reflect other sources of exposure (e.g., occupational exposure) or distance to agricultural land within the county. Agricultural pesticide use at the county level may not reflect dietary exposure due to widespread distribution of food across regions, potentially leading to exposure misclassification and attenuation of observed associations. Additionally, residual confounding is possible as we were limited to data on county-level factors and were unable to adjust for all known breast cancer risk factors. However, our findings of minimal spatial dependence in model residuals suggest that unmeasured spatially structured confounding is unlikely to explain the observed associations. Finally, exclusion of counties with missing data may introduce bias, though differences in county characteristics between those included and excluded from analysis were minimal in magnitude. Future research incorporating more detailed and longitudinal data is needed to better capture individual-level pesticide exposures and breast cancer risk over time. Studies integrating biomarkers of exposure and genetic susceptibility would provide stronger evidence of causality and help clarify underlying biological mechanisms.

## Conclusion

Our findings suggest modest positive associations between county-level endocrine-disrupting pesticides and breast cancer incidence in rural U.S. counties, with variation across pesticide types. These findings underscore the need for rigorous longitudinal studies on pesticide-related breast cancer risks, as well as for targeted monitoring and tailored public health interventions.

## Supplementary Information

Below is the link to the electronic supplementary material.

Supplementary file1 (DOCX 2973 KB)

## Data Availability

The datasets analyzed for this study are publicly available from the USGS Pesticide National Synthesis Project and the National Cancer Institute’s State Cancer Profiles.
