# Supplementary material for: Endocrine-disrupting pesticides and breast cancer incidence in the United States: an ecological analysis
Source: Cancer Causes Control. 2026 Jun 1;37(7):104. doi: 10.1007/s10552-026-02188-3 (PMC13226314; doi:10.1007/s10552-026-02188-3)
Supplement: Supplementary file 1 — Supplementary file1 (DOCX 2973 KB) [file 10552_2026_2188_MOESM1_ESM.docx]

**Supplementary Figure 1.** Study sample

**3,143**  Total counties in the United States of America

**3,066** Counties with pesticide data

**77**  Missing data on pesticides used during 2001 - 2015

**609**  Missing average age-adjusted breast cancer incidence rates for the period 2016 – 2020

**397**  Data not available

**212**  Data has been suppressed to ensure confidentiality and stability of rate estimates

**2,457** Counties included in the analysis

**Supplementary Figure 2.** Trends in the amount of endocrine-dirsupting pesticides used in kilograms across rural and urban counties in the U.S. by class over the period 2001-2015.


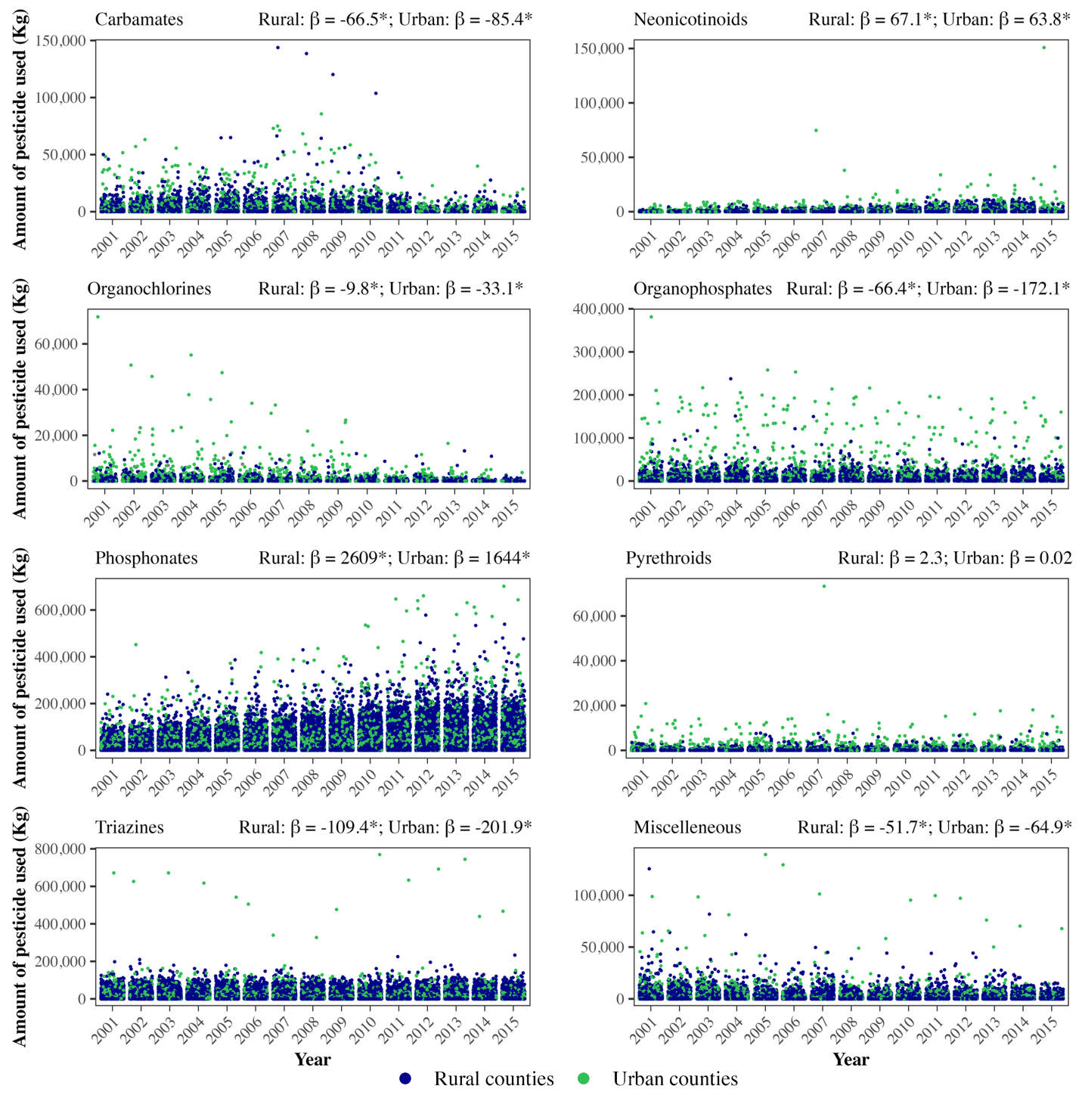


Note: Beta estimates represent the average annual change in pesticide use (kg per year) for each pesticide class, obtained from linear regression of pesticide use on year, along with the corresponding p-values.

*Significant p-values, p-value<0.001

**Supplementary Figure 3. (A)** Average annual total of pesticides with endocrine-disruption properties used in kilograms (Kg) from 2001 to 2015 and **(B)** Average age-adjusted breast cancer incidence rates per 100,000 during 2016 – 2020 in the U.S. Counties.


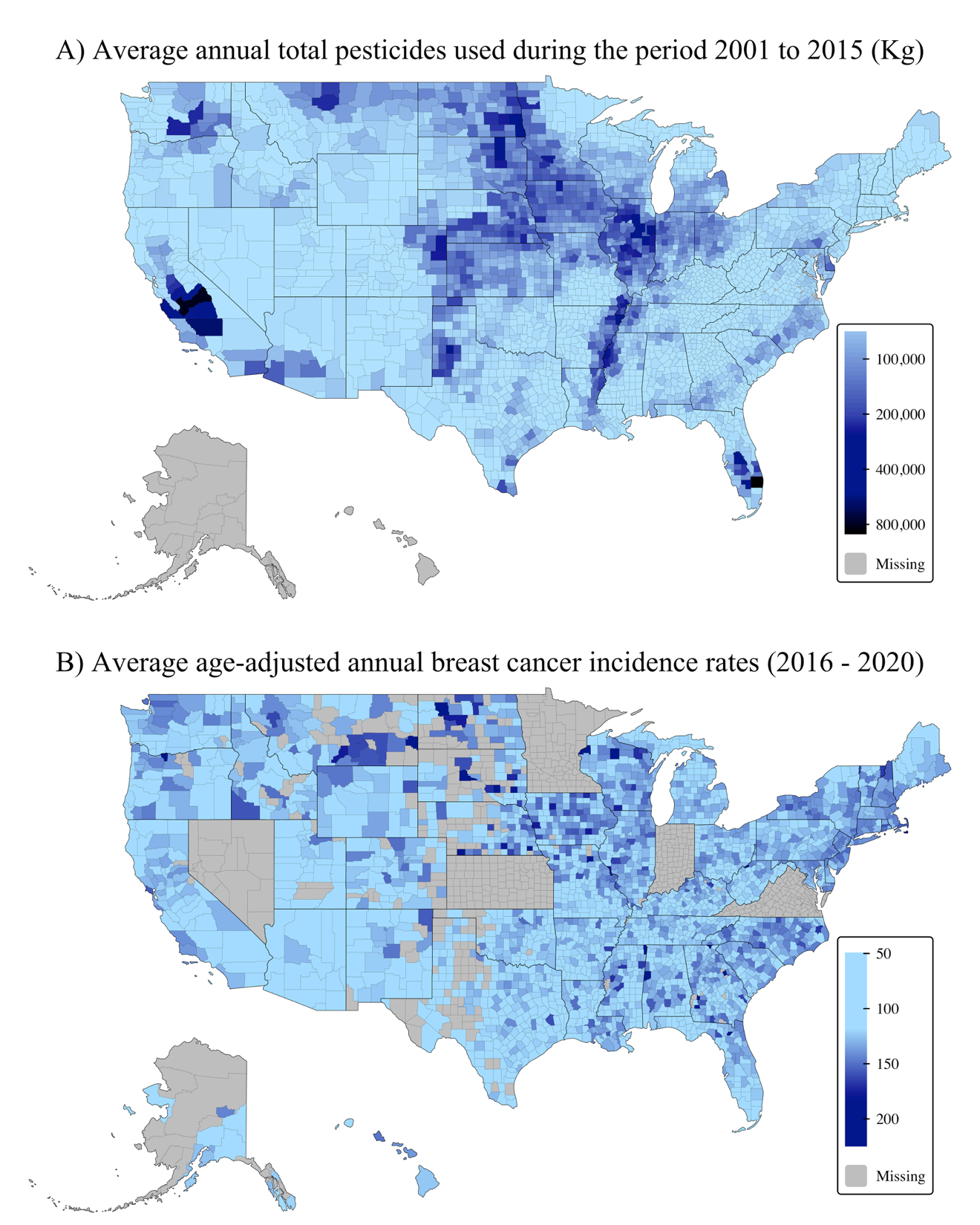


*Darker blue corresponds to higher values, and lighter blue corresponds to lower values, for both pesticide use and cancer rates.

**Supplementary Figure 4.** Local Indicators of Spatial Association (LISA) clusters of breast cancer incidence and county-level pesticide classes in the U.S.


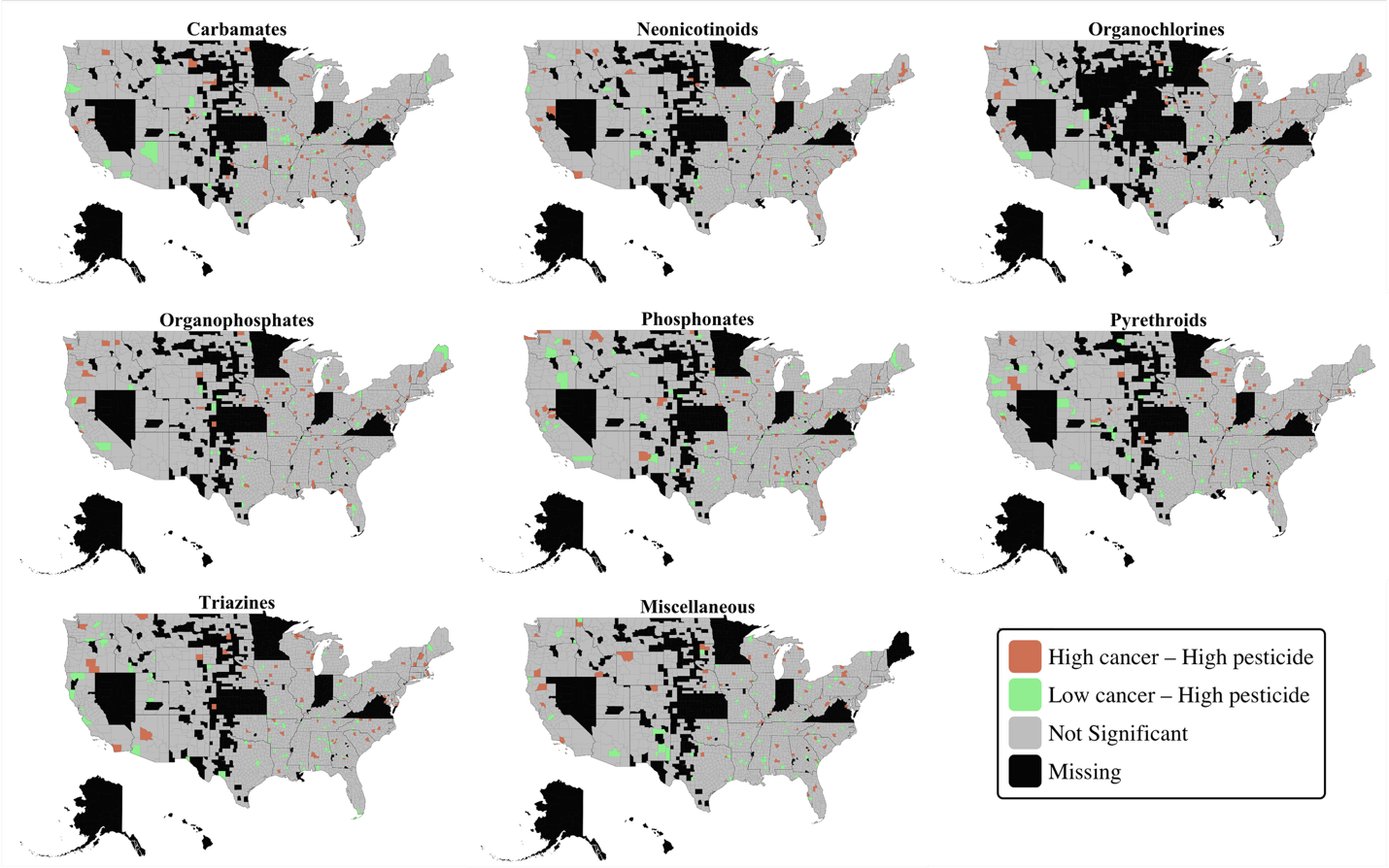


*Counties are classified as High-High, Low-Low, High-Low, or Low-High based on the local bivariate Moran’s I (LISA) between breast cancer incidence and each pesticide class. High-High counties (red) represent areas with above-average incidence surrounded by counties with above-average pesticide use; Low-Low represent below-average incidence surrounded by below-average pesticide use; High-Low and Low-High (green) indicate spatial mismatches between the two variables. Statistical significance was assessed using Monte Carlo permutations. Only statistically significant locations (p < 0.05) are classified; all others are treated as not significant (grey) and are not interpreted as clusters. Results are shown separately for each of the eight pesticide classes.

*Some counties were excluded from the LISA analysis due to missing data for the outcome (black), exposure, or covariates, or because they had no neighbors under queen contiguity.

**Supplementary Table 1.** County-level characteristics of counties included in the analysis and those excluded due to missing breast cancer rates

| **Characteristics** | **Analytic Sample**  **N = 2,457** | **Excluded Sample**  **N = 609** | **Effect size^g^ (95% CI)** | **p-value^h^** |
| --- | --- | --- | --- | --- |
|  | **Median**  **(IQR)** | **Median**  **(IQR)** |  |  |
| ***Pesticide use*** |  |  |  |  |
| Total pesticide | 15947.2 (64379.4) | 43224.1 (86881.7) | -0.19  (-0.27, -0.10) | <0.001 |
| Carbamates | 234.4 (547.0) | 179.9 (312.0) | 0.23  (0.14, 0.32) | <0.001 |
| Neonicotinoids | 151.6 (572.6) | 299.1 (717.6) | -0.03  (-0.12, 0.06) | <0.001 |
| Organochlorines | 26.7 (95.4) | 20.3 (58.7) | 0.14  (0.04, 0.24) | <0.001 |
| Organophosphates | 1267.7 (2886.8) | 1749.2 (2580.5) | 0.11  (0.02, 0.20) | <0.001 |
| Phosphonates | 8483.3 (39426.0) | 28756.6 (55459.1) | -0.24  (-0.33, -0.15) | <0.001 |
| Pyrethroids | 56.2 (161.4) | 58.7 (107.3) | 0.15  (0.06, 0.23) | 0.426 |
| Triazines | 2625.1 (12491.5) | 7226.1 (23646.8) | -0.18  (-0.27, -0.09) | <0.001 |
| Miscellaneous | 491.5 (984.4) | 500.1 (624.6) | 0.14  (0.05, 0.23) | 0.376 |
| ***Covariates*** | **Mean**  **(SD)** | **Mean**  **(SD)** |  |  |
| % Smoking^a^ | 18.1 (3.5) | 16.8 (3.1) | 0.40  (0.31, 0.49) | <0.001 |
| % Poverty^b^ | 19.5 (6.9) | 16.2 (6.4) | 0.48  (0.39, 0.57) | <0.001 |
| % Unemployed^c^ | 6.3 (2.5) | 4.6 (2.9) | 0.65  (0.56, 0.74) | <0.001 |
| % No high school diploma^d^ | 13.7 (6.0) | 12.1 (6.8) | 0.26  (0.17, 0.35) | <0.001 |
| % Uninsured^e^ | 10.6 (4.8) | 10.3 (5.4) | 0.06  (-0.03, 0.15) | 0.003 |
| % Residential mobility^f^ | 12.6  (4.0) | 11.6  (4.5) | 0.25  (0.16, 0.34) | <0.001 |

^a^Proportion of adults in the county that report current smoking

^b^Percentage of the county population living below the federal **poverty line**

**^c^**Percentage of the county’s **labor force (age 16+ civilians)** that is actively looking for work but is currently unemployed

^d^Proportion of adults (age 25+) in the county with no high school diploma

^e^Percentage of adults uninsured in the total noninstitutionalized population

^f^Percentage of residents living in a different residence one year prior

^g^Cohen’s d was calculated for continuous variables to quantify the standardized mean differences between groups

^h^P-value correspond to the wilcoxon-rank sum test comparing pesticides and covariates between the analytic and excluded samples

**Supplementary Table 2.** Age-adjusted and multivariable-adjusted associations between breast cancer incidence and total pesticide use (categorized into tertiles) in the U.S., overall and by rurality.

| **Pesticide use (Kg)**  **(2001 – 2015)** | **Age-adjusted breast cancer rates (2016 – 2020)** | | | | | |
| --- | --- | --- | --- | --- | --- | --- |
|  | **Overall U.S.**  **N = 2,457** | | **Rural Counties**  **N = 1,472** | | **Urban Counties**  **N = 985** | |
|  | **Age adjusted**  $\boldsymbol{RR}$*****  **(95% CI)** | **Multivariable**  $\boldsymbol{aRR}$******  **(95% CI)** | **Age adjusted**  $\boldsymbol{RR}$*****  **(95% CI)** | **Multivariable**  $\boldsymbol{aRR}$******  **(95% CI)** | **Age adjusted**  $\boldsymbol{RR}$*****  **(95% CI)** | **Multivariable**  $\boldsymbol{aRR}$******  **(95% CI)** |
| **Total pesticide** |  |  |  |  |  |  |
| Low (<5,600) | 1.00 (Ref) | 1.00 (Ref) | 1.00 (Ref) | 1.00 (Ref) | 1.00 (Ref) | 1.00 (Ref) |
| Medium (≥5,600 & <42,000) | 1.01 [1.00, 1.03] | 1.02 [1.00, 1.03] | 1.02 [1.00, 1.04] | 1.03 [1.00, 1.05] | 0.99 [0.97, 1.01] | 0.99 [0.97, 1.01] |
| High (≥42,000) | **1.04 [1.02, 1.05]** | **1.03 [1.01, 1.04]** | **1.07 [1.04, 1.09]** | **1.06 [1.03, 1.08]** | 1.00 [0.98, 1.02] | 0.99 [0.97, 1.01]] |

Abbreviations: CI, confidence interval; p-value_int_, rural urban interaction p-value;

*$RR$ = Rate ratio per IQR increase in pesticide use.

**$aRR$ = Adjusted rate ratio per IQR increase in pesticide use; Multivariable model adjusted for % smoking, % poverty, % unemployed, % no high school diploma, % uninsured, and % residential mobility.

**Supplementary Table 3.** Decriptive statistics and multivariable-adjusted associations between breast cancer incidence and individual pesticide use (38 pesticides analyzed separately) per IQR increase in the U.S., overall and in rural counties.

| **Pesticide type**  **(2001 – 2015)** | **Counties with pesticide use (2001-2015)** | **Age-adjusted breast cancer rates (2016 – 2020)** | | | |
| --- | --- | --- | --- | --- | --- |
|  |  | **Overall U.S.** | | **Rural Counties** | |
|  |  | **Median pesticide use (IQR)** | **Multivariable**  $\boldsymbol{aRR}$**^a^**  **(95% CI)** | **Median pesticide use (IQR)** | **Multivariable**  $\boldsymbol{aRR}$**^a^**  **(95% CI)** |
| ***Carbamates*** |  |  |  |  |  |
| Aldicarb | 1770 | 101 (568) | 1.00 [1.00, 1.00] | 100 (575) | 1.00 [1.00, 1.01] |
| Carbaryl | 3042 | 160 (341) | 1.00 [1.00, 1.00] | 171 (347) | 1.00 [1.00, 1.01] |
| Lindane | 2559 | 4 (7) | 1.00 [1.00, 1.01] | 4 (8) | 1.00 [1.00, 1.01] |
| Propoxur^b^ | 4 | 108  (329) | - | 121  (342) | - |
| ***Neonicotinoids*** |  |  |  |  |  |
| Acetamiprid | 2218 | 1 (5) | 1.00 [1.00, 1.00] | 1 (3) | 1.00 [1.00, 1.00] |
| Clothianidin | 2971 | 51 (269) | 1.00 [1.00, 1.00] | 57 (338) | 1.01 [1.00, 1.02] |
| Dinotefuran | 1277 | 2 (6) | 1.00 [1.00, 1.00] | 2 (7) | 1.00 [1.00, 1.00] |
| Imidacloprid | 3033 | 61 (171) | 1.00 [1.00, 1.00] | 71 (183) | 1.01 [1.00, 1.02] |
| Thiacloprid | 840 | 1 (4) | 1.00 [1.00, 1.00] | 1 (3) | 1.00 [1.00, 1.00] |
| Thiamethoxam | 3013 | 44 (158) | 1.01 [1.00, 1.01] | 53 (181) | **1.02^†^ [1.01, 1.02]** |
| ***Organochlorines*** |  |  |  |  |  |
| Dicofol | 1293 | 12 (67) | 1.00 [1.00, 1.00] | 13 (77) | 1.00 [1.00, 1.01] |
| Endosulfan | 2733 | 18 (64) | 1.00 [1.00, 1.00] | 14 (57) | 1.00 [1.00, 1.00] |
| Methoxychlor | 507 | 10 (89) | 1.00 [1.00, 1.01] | 14 (90) | 1.00 [0.98, 1.02] |
| ***Organophosphates*** |  |  |  |  |  |
| Azinphos-Methyl | 2491 | 14 (43) | 1.00 [1.00, 1.00] | 12 (31) | 1.00 [1.00, 1.00] |
| Bensulide | 1980 | 13 (41) | 1.00 [1.00, 1.00] | 10 (29) | 1.00 [1.00, 1.00] |
| Chlorpyrifos | 3053 | 689 (1564) | 1.00 [1.00, 1.00] | 750 (1655) | **1.01^†^ [1.01, 1.02]** |
| Diazinon | 2970 | 19 (50) | 1.00 [1.00, 1.00] | 16 (44) | 1.00 [1.00, 1.00] |
| Dicrotophos | 863 | 76 (340) | 1.00 [1.00, 1.01] | 85 (392) | 1.00 [1.00, 1.01] |
| Dimethoate | 2989 | 121 (308) | 1.00 1.00, 1.00] | 136 (338) | 1.00 1.00, 1.01] |
| Disulfoton | 2209 | 39 (160) | 1.00 1.00, 1.00] | 48 (192) | 1.00 1.00, 1.00] |
| Ethoprophos | 1374 | 16 (143) | 1.000 [1.00, 1.00] | 24 (186) | 1.00 [1.00, 1.00] |
| Fenamiphos | 792 | 20 (116) | 1.00 [1.00, 1.01] | 23 (124) | 1.02 [1.00, 1.04] |
| Malathion | 3040 | 108 (279) | 1.00 [1.00, 1.00] | 127 (294) | 1.00 [1.00, 1.01] |
| Naled | 511 | 36 (396) | 1.00 1.00, 1.00] | 152 (568) | 1.00 1.00, 1.01] |
| Parathion^b^ | 36 | 13  (49) | - | 9  (31) | - |
| Phorate | 2674 | 117 (403) | 1.00 [1.00, 1.00] | 142 (457) | 1.00 [1.00, 1.01] |
| ***Phosphonates*** |  |  |  |  |  |
| Glufosinate | 2984 | 146 (608) | 1.00 [1.00, 1.01] | 173 (716) | **1.01^†^ [1.01, 1.02]** |
| Glyphosate | 3068 | 11306 (44550) | 1.01 [1.00, 1.01] | 13311 (54262) | **1.02^†^ [1.01, 1.03]** |
| ***Pyrethroids*** |  |  |  |  |  |
| Cypermethrin | 1354 | 9 (53) | 1.00 [1.00, 1.01] | 10 (64) | 1.01 [1.00, 1.01] |
| Fenpropathrin | 1578 | 2 (8) | 1.00 [1.00, 1.00] | 2 (5) | 1.00 [1.00, 1.00] |
| Permethrin | 3035 | 37 (119) | 1.00 [0.997, 1.001] | 37 (121) | 1.01 [1.00, 1.02] |
| ***Triazines*** |  |  |  |  |  |
| Atrazine | 3035 | 2670 (13362) | 1.00 [1.00, 1.01] | 2824 (15633) | 1.01 [1.00, 1.02] |
| Propozine | 467 | 50 (270) | 1.00 [1.00, 1.01] | 58 (297) | 1.00 [0.99, 1.02] |
| Simazine | 2811 | 383 (1354) | 1.00 [1.00, 1.00] | 358 (1301) | 1.00 [1.00, 1.01] |
| ***Miscellaneous*** |  |  |  |  |  |
| Fosetyl | 926 | 6 (28) | 1.00 [1.00, 1.00] | 4 (16) | 1.00 [1.00, 1.00] |
| Terbufos | 2818 | 158 (515) | 1.00 [1.00, 1.01] | 165 (581) | 1.00 [1.00, 1.01] |
| Tribufos | 776 | 366 (1520) | 1.00 [1.00, 1.01] | 412 (1587) | 1.01 [1.00, 1.01] |
| Triclopyr | 3007 | 108 (329) | 1.00 [0.99, 1.00] | 121 (342) | 1.00 [0.99, 1.00] |

Abbreviations: IQR, Interquartile range; CI, confidence interval;

^a^$aRR$ = Adjusted rate ratio per IQR increase in pesticide use; Multivariable model adjusted for % smoking, % poverty, % unemployed, % no high school diploma, % uninsured, and % residential mobility.

^b^Estimates could not be computed for some ingredients due to limited sample sizes

^†^Statistically significant after controlling for multiple comparisons using the Benjamini-Hochberg false discovery rate (FDR < 0.05)

**Supplementary Table 4.** Age-adjusted and multivariable-adjusted associations between breast cancer incidence and interquartile range (IQR) increase in county-level pesticide use in the U.S., overall and by rurality using EPEST “low” estimates

| **Pesticide use (Kg)**  **(2001 – 2015)** | **Age-adjusted breast cancer rates (2016 – 2020)** | | | | | |
| --- | --- | --- | --- | --- | --- | --- |
|  | **Overall U.S.**  **N = 2,457** | | | **Rural Counties**  **N = 1,472** | | |
|  | **Median pesticide use (IQR)** | **Age adjusted**  $\boldsymbol{RR}$*****  **(95% CI)** | **Multivariable**  $\boldsymbol{aRR}$******  **(95% CI)** | **Median pesticide use (IQR)** | **Age adjusted**  $\boldsymbol{RR}$*****  **(95% CI)** | **Multivariable**  $\boldsymbol{aRR}$******  **(95% CI)** |
| Total pesticide | 16,114 (59,391) | 1.01 [1.00, 1.01] | 1.01 [0.999, 1.01] | 17,154 (68,893) | **1.03 [1.02, 1.04]** | **1.02 [1.01, 1.03]** |
| Carbamates | 68 (287) | 1.00 [1.00, 1.00] | 1.00 [1.000, 1.00] | 57 (273) | 1.00 [1.00, 1.00] | 1.00 [1.00, 1.00] |
| Neonicotinoids | 123 (514) | 1.00 [1.00, 1.01] | 1.00 [1.00, 1.01] | 130 (599) | **1.02 [1.01, 1.03]** | 1.01 [1.00, 1.02] |
| Organochlorines | 12 (43) | 1.00 [1.00, 1.00] | 1.00 [1.00, 1.00] | 10 (36) | 1.00 [1.00, 1.00] | 1.00 [1.00, 1.00] |
| Organophosphates | 512 (1,616) | 1.00 [1.00, 1.00] | 1.00 [1.00, 1.00] | 496 (1,592) | 1.00 [1.00, 1.01] | 1.01 [1.00, 1.01] |
| Phosphonates | 7,918 (38,535) | 1.01 [1.00, 1.02] | 1.01 [1.00, 1.01] | 9,116 (47,991) | **1.03 [1.02, 1.04]** | **1.02 [1.01, 1.03]** |
| Pyrethroids | 18 (59) | 1.00 [1.00, 1.000] | 1.00 [1.00, 1.00] | 15 (58) | 1.00 [1.00, 1.00] | 1.00 [1.00, 1.004] |
| Triazines | 2,173 (11,608) | 1.01 [1.00, 1.01] | 1.00 [1.00, 1.01] | 2,118 (13,536) | **1.02 [1.01, 1.02]** | 1.01 [1.00, 1.02] |
| Miscellaneous | 305 (894) | 1.00 [1.00, 1.000] | 1.00 [1.00, 1.00] | 339 (950) | 1.00 [1.00, 1.00] | 1.01 [1.00, 1.01] |

Abbreviations: IQR, Inter quartile range; CI, confidence interval;

*$RR$ = Rate ratio per IQR increase in pesticide use.

**$aRR$ = Adjusted rate ratio per IQR increase in pesticide use; Multivariable model adjusted for % smoking, % poverty, % unemployed, % no high school diploma, % uninsured, and % residential mobility.

**Supplementary Table 5.** Age-adjusted and multivariable-adjusted associations between county-level pesticide use (per interquartile range (IQR) increase) and young -onset breast cancer incidence (<50 years) in the U.S., overall and in rural counties.

| **Pesticide use (Kg)**  **(2001 – 2015)** | **Young-onset age-adjusted breast cancer rates (2017 – 2021)** | | | | **p-value_int_** |
| --- | --- | --- | --- | --- | --- |
|  | **Overall U.S.**  **N = 1,405** | | **Rural counties**  **N = 548** | |  |
|  | **Age adjusted**  $\boldsymbol{RR}$*****  **(95% CI)** | **Multivariable**  $\boldsymbol{aRR}$******  **(95% CI)** | **Age adjusted**  $\boldsymbol{RR}$*****  **(95% CI)** | **Multivariable**  $\boldsymbol{aRR}$******  **(95% CI)** |  |
| Total pesticide | 1.00 [0.99, 1.01] | 1.00 [0.99, 1.00] | 1.01 [0.99, 1.03] | 1.01 [0.99, 1.03] | 0.18 |
| Carbamates | 1.00 [1.00, 1.00] | 1.00 [1.00, 1.00] | 1.00 [1.00, 1.00] | 1.00 [1.00, 1.01] | 0.26 |
| Neonicotinoids | 1.00 [0.99, 1.00] | 1.00 [0.99, 1.00] | 1.01 [0.99, 1.03] | 1.01 [0.99, 1.02] | 0.22 |
| Organochlorines | 1.00 [1.00, 1.00] | 1.00 [1.00, 1.00] | 1.00 [1.00, 1.00] | 1.00 [1.00, 1.00] | 0.81 |
| Organophosphates | 1.00 [0.99, 1.00] | 1.00 [0.99, 1.00] | 0.99 [0.98, 1.01] | 1.00 [0.98, 1.01] | 0.98 |
| Phosphonates | 1.00 [0.99, 1.01] | 1.00 [0.99, 1.01] | 1.01 [1.00, 1.03] | 1.01 [0.99, 1.03] | 0.09 |
| Pyrethroids | 1.00 [0.99, 1.00] | 1.00 [0.99, 1.00] | 1.00 [0.98, 1.01] | 0.99 [0.98, 1.01] | 0.93 |
| Triazines | 1.00 [1.00, 1.01] | 1.00 [0.99, 1.00] | 1.01 [0.99, 1.02] | 1.00 [0.98, 1.02] | 0.77 |
| Miscellaneous | 1.00 [1.00, 1.00] | 1.00 [1.00, 1.00] | 1.00 [1.00, 1.01] | 1.01 [1.00, 1.02] | 0.01 |

Abbreviations: CI, confidence interval; p-value_int_, rural urban interaction p-value after adjusting for covariates;

*$RR$ = Rate ratio per IQR increase in pesticide use.

**$aRR$ = Adjusted rate ratio per IQR increase in pesticide use; Multivariable model adjusted for % smoking, % poverty, % unemployed, % no high school diploma, % uninsured, and % residential mobility.
